# Acute Hemodynamic Responses to Enhanced External Counterpulsation in Patients With Coronary Artery Disease

**DOI:** 10.3389/fcvm.2021.721140

**Published:** 2021-11-12

**Authors:** Yahui Zhang, Ziqi Chen, Zhouming Mai, Wenjuan Zhou, Hui Wang, Xiaodong Zhang, Wenbin Wei, Jianhang Du, Guifu Wu

**Affiliations:** ^1^Department of Cardiology, The Eighth Affiliated Hospital, Sun Yat-sen University, Shenzhen, China; ^2^National Health Commission (NHC) Key Laboratory of Assisted Circulation, Sun Yat-sen University, Guangzhou, China; ^3^Guangdong Innovative Engineering and Technology Research Center for Assisted Circulation, Shenzhen, China; ^4^Department of Cardiology, Huizhou Third People's Hospital, Huizhou, China; ^5^Department of Cardiac Ultrasound, The Eighth Affiliated Hospital, Sun Yat-sen University, Shenzhen, China; ^6^Department of Physical Education, Nanjing University of Finance and Economics, Nanjing, China

**Keywords:** enhanced external counterpulsation, carotid arteries, peripheral hemodynamics, coronary artery disease, acute response

## Abstract

**Purpose:** Enhanced external counterpulsation is a non-invasive treatment that increases coronary flow in patients with coronary artery disease (CAD). However, the acute responses of vascular and blood flow characteristics in the conduit arteries during and immediately after enhanced external counterpulsation (EECP) need to be verified.

**Methods:** Forty-two patients with CAD and 21 healthy controls were recruited into this study to receive 45 min-EECP. Both common carotid arteries (CCAs), namely, the left carotid (LC) and right carotid (RC), the right brachial (RB), and right femoral (RF) artery were imaged using a Color Doppler ultrasound. The peak systolic velocity (PSV), end-diastolic velocity (EDV), mean inner diameter (ID), resistance index (RI), and mean flow rate (FR) were measured and calculated before, during, and after the 45 min-EECP treatment.

**Results:** During EECP, in the CCAs, the EDV was significantly decreased, while the RI was markedly increased in the two groups (both *P* < 0.01). However, immediately after EECP, the RI in the RC was significantly lower than that at the baseline in the patients with CAD (*P* = 0.039). The FR of the LC was markedly increased during EECP only in the CAD patients (*P* = 0.004). The PSV of the patients with CAD was also significantly reduced during EECP (*P* = 0.015) and immediately after EECP (*P* = 0.005) compared with the baseline. Moreover, the ID of the LC, RB, and RF was significantly higher immediately after EECP than that at the baseline (all *P* < 0.05) in the patients with CAD. In addition, they were also higher than that in the control groups (all *P* < 0.05). Furthermore, by the subgroup analysis, there were significant differences in the FR, PSV, and RI between females and males during and immediately after EECP (all *P* < 0.05).

**Conclusions:** Enhanced external counterpulsation creates different responses of vascular and blood flow characteristics in carotid and peripheral arteries, with more significant effects in both the carotid arteries. Additionally, the beneficial effects in ID, blood flow velocity, RI, and FR after 45 min-EECP were shown only in the patients with CAD. More importantly, acute improvement of EECP in the FR of the brachial artery was showed in males, while the FR and RI of the carotid arteries changed in females.

## Introduction

Enhanced external counterpulsation is a non-invasive treatment for patients with stable coronary artery disease (CAD), who are not amenable to standard revascularization procedures, such as percutaneous coronary intervention (1, 2). Several studies have demonstrated that enhanced external counterpulsation (EECP) alleviates symptoms of angina and reduces myocardial ischemia (3, 4). Enhanced external counterpulsation also significantly increased myocardial perfusion and coronary collateral flow in patients with CAD (5, 6). In addition, EECP has been previously shown to increase coronary artery flow velocity (7). However, little is known about whether it can affect vascular and blood flow characteristic variables in conduit arteries such as the carotid and those of the limbs, as the pathogenesis and progression of CAD are influenced not only by the behavior of the coronary arteries but also by other more peripheral systemic vessels (8).

These alterations of peripheral vascular and blood flow characteristics play a key role in the modulation of central arterial function, cardiac load, and myocardial perfusion (9). For instance, lower peak systolic velocity (PSV), higher resistance index (RI), and lower end-diastolic velocity (EDV) in the common carotid artery (CCA) are related to adverse cardiovascular events (10–12). Studies showed that the left and right CCA may exhibit different prognostic values in the investigated population (13, 14). Due to the different anatomical origins of the left and the right CCA, it was speculated that hemodynamics would have different effects depending on whether the left carotid (LC) or right carotid (RC) artery was considered (15). Brachial artery blood flow velocities are associated with carotid atherosclerosis (16). More importantly, the presence of atherosclerotic plaques and altered vascular function in the aorta, femoral, and carotid arteries are strong predictors of CAD (17).

Nevertheless, there have been few studies on the effects of EECP on conduit artery vascular and blood flow characteristics, and the controversy remains even in patients with CAD. It has been reported that EECP can increase the peak limb blood flow and improve the endothelium-dependent vasodilation in the resistance arteries (18). A similar improvement has been shown in the femoral and brachial arteries (19). Enhanced external counterpulsation can also reduce carotid vascular resistance (9). Whereas, others have found that EECP does not affect the central and peripheral arterial stiffness (20). It has also been reported that the cerebral mean flow velocities increased in patients with stroke during EECP, while it remained unchanged in the elderly controls (21).

Given the controversial reports of the effects of EECP on systemic circulation, this study aimed to investigate the acute effects of EECP on the vascular and blood flow characteristics in the carotid, brachial, and femoral arteries in patients with CAD. Meanwhile, to further explore the mechanisms of these effects, we also aimed to compare the responses to those of the controls at the baseline, during, and immediately after 45 min-EECP.

## Materials and Methods

### Participants

We enrolled patients with CAD to EECP treatment, diagnosed by angiographically proven stenosis ≥50% in at least one major coronary artery. They were admitted to the Department of Cardiovascular Medicine of the Eighth Affiliated Hospital of Sun Yat-sen University. The exclusion criteria were contraindications for EECP, including uncontrolled hypertension (SBP ≥ 180 mmHg and/or DBP ≥ 100 mmHg), carotid dissection, and deep vein thrombosis at lower extremities. In addition, we recruited healthy people as the control through the Health Examination Center of the Eighth Affiliated Hospital of Sun Yat-sen University. After explaining the purpose of this study and the potential risks, all the participants provided written informed consent. This study was conducted in accordance with the Declaration of Helsinki and was approved by the medical ethics committee of the Eighth Affiliated Hospital of Sun Yat-sen University.

### Procedures

All participants received a single, 45-min session of EECP treatment. They lay supine on the EECP treatment bed with their legs and buttocks wrapped in cuffs, which were sequentially inflated from the lower to the upper thigh and buttocks at the beginning of the diastolic phase, followed by a quick, simultaneous deflation of all the cuffs just before the onset of systole. The EECP was conducted using an Oxygen Saturation Monitoring Enhanced External Counterpulsation Instrument (PSK P-ECP/TM, Chongqing, China). The pressure generated by the device was 0.028–0.033 MPa (212.8–250.8 mmHg).

### Measurement of Peripheral Vascular and Blood Flow Characteristics

A Color Doppler Ultrasound (GE logic E) examination was performed 10 min before the start of the EECP at 15–25 min after its start and 30 s−1 min immediately after the end of the session. The measurement time for each part was about 2 min and the measurements were performed in the order: RC, LC, right brachial (RB), and right femoral (RF) arteries (19, 22). The right and left CCAs were scanned 1.5 cm proximal to the internal-external carotid bifurcation. The RB and RF measurement sites were fixed approximately 5 cm above the antecubital fossa and 2 cm below the inguinal ligament, respectively. The RF artery was not measured during the EECP because the legs and buttocks on this side were wrapped in the cuffs.

### Variables Calculation

The variables, PSV, RI, peak diastolic velocity (VD), PSV/VD (VS/VD), and velocity-time integral (VTI) in the CCAs were continuously recorded for 10 s and then were tracked and calculated.

The mean inner diameters (ID) of all the arteries were calculated as


(1) $$\begin{array}{l} \overset{-}{ID}=\left(ID_{dia}+ID_{sys}\right)/2, \end{array}$$

where *ID*_*sys*_ and *ID*_*dia*_ are the systolic and diastolic diameters, respectively.

The *RI* was analyzed only in the carotid arteries because, as a measure of cerebral resistance, it is closely associated with cardiovascular risk. It was calculated as


(2) $$\begin{array}{l} RI=\left(V_{peak}-V_{end-dia}\right)/V_{peak}, \end{array}$$

where V_*peak*_ is the PSV and V_*end*−*dia*_ is the EDV.

The mean flow rate (FR) was calculated from the vessel diameter, cardiac period, and velocity-time integral as


(3) $$\begin{array}{l} \overset{-}{FR}=\left(\frac{1}{4}\pi \overset{-}{\left(ID\right)}\times VTI\right)/t \end{array}$$

where VTI is the averaged velocity-time integral, and *t* is the averaged cardiac cycle time.

### Statistical Analysis

The results are expressed as means ± SE. The normal distribution for all the vascular and blood flow characteristic variables was evaluated by a Kolmogorov–Smirnov test. The basic characteristics of the two groups were compared by an independent *t*-test. A Chi-square test was also used for the analysis of the gender and risk factors in the two groups. The dependent variables before, during, and after the 45-min EECP, and between the patients with CAD and controls were analyzed by two repeated measures-ANOVA. Gender was regarded as a covariate in the two-way ANOVA of the inter and intra groups. In addition, the hemodynamic variables in each artery were also performed by subgroup analysis of gender. The *post hoc* analysis was conducted using Fisher's least significant difference. The software SPSS version 20.0 (IBM SPSS Statistics, Armonk, New York, United States) was used for all the statistical tests, and *P* < 0.05 was taken as a measure of statistical significance.

## Results

The basic information of the two groups is listed are Table 1. There were 42 patients with CAD and 21 healthy controls in the study. There were no significant differences between them in number, age, height, weight, and body mass index (BMI) (*p* > 0.05), while significant differences appeared in gender and risk factors in this study (*P* < 0.05).

**Table 1:** Base information and major cardiovascular risk factors in the two groups.

**Variables**	**CAD**	**Control**	***P*-value**
Number	42	21	
Age (year)	57.52 ± 9.24	53.95 ± 8.44	0.142
Female (percentage/*n*)	33.3 (14)	66.67 (14)	0.012
Height (cm)	164.90 ± 8.37	161.25 ± 9.47	0.129
Weight (kg)	69.21 ± 11.60	65.30 ± 13.31	0.234
BMI (kg/m^2^)	25.34 ± 2.95	24.36 ± 3.08	0.245
Hypertension (percentage/*n*)	64.29 (27)	0(0)	0.000
Hyperlipidemia (percentage/*n*)	47.62 (20)	0(0)	0.000
Hyperglycemia (percentage/*n*)	40.48 (17)	0(0)	0.000
Smoking (percentage/*n*)	28.57 (12)	0(0)	0.000

**Figure 1:**
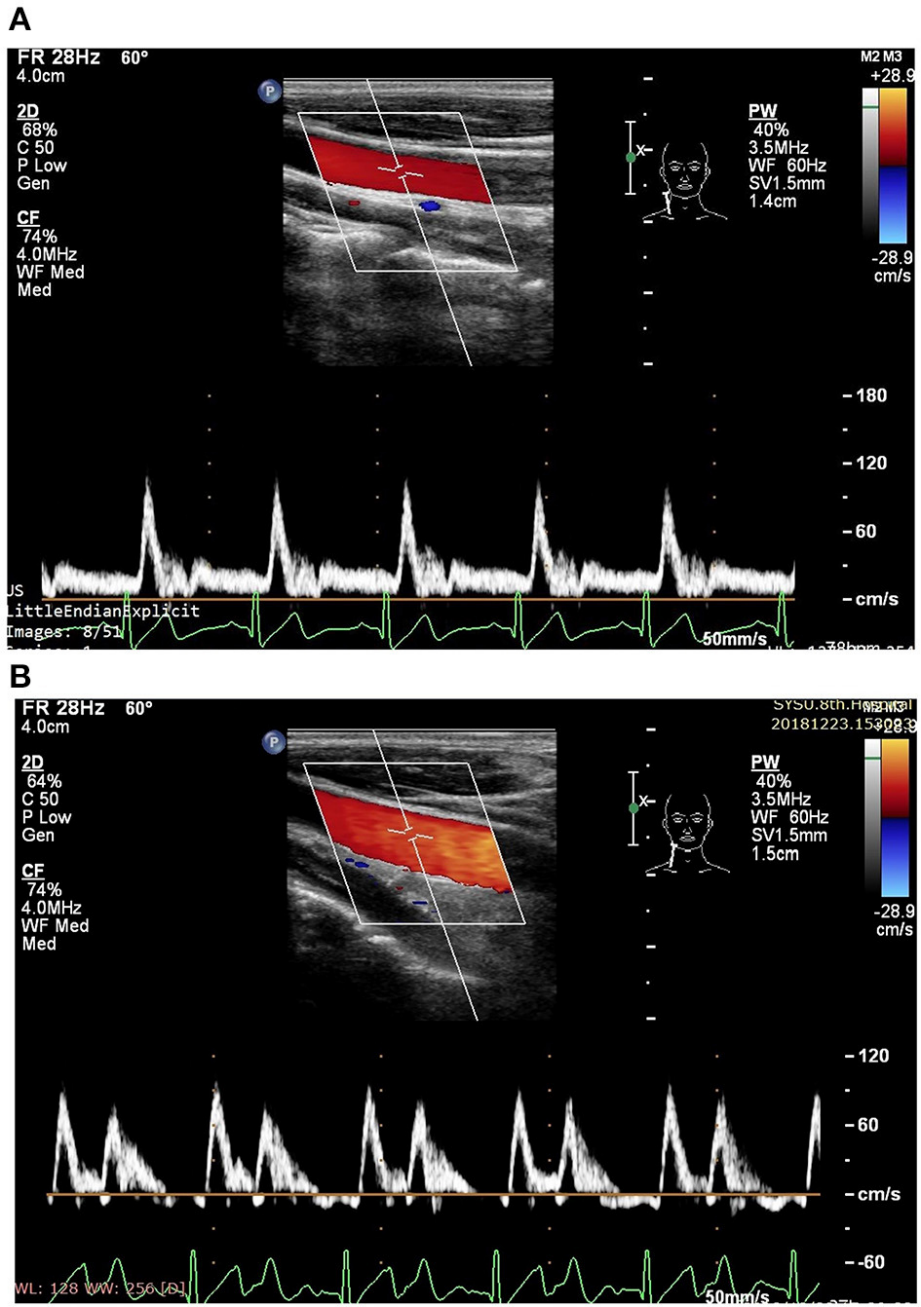
Measurement of blood flow velocity spectrum in the right common carotid artery (CCA) [**A** before enhanced external counterpulsation (EECP) and **B** during EECP].

**Figure 2:**
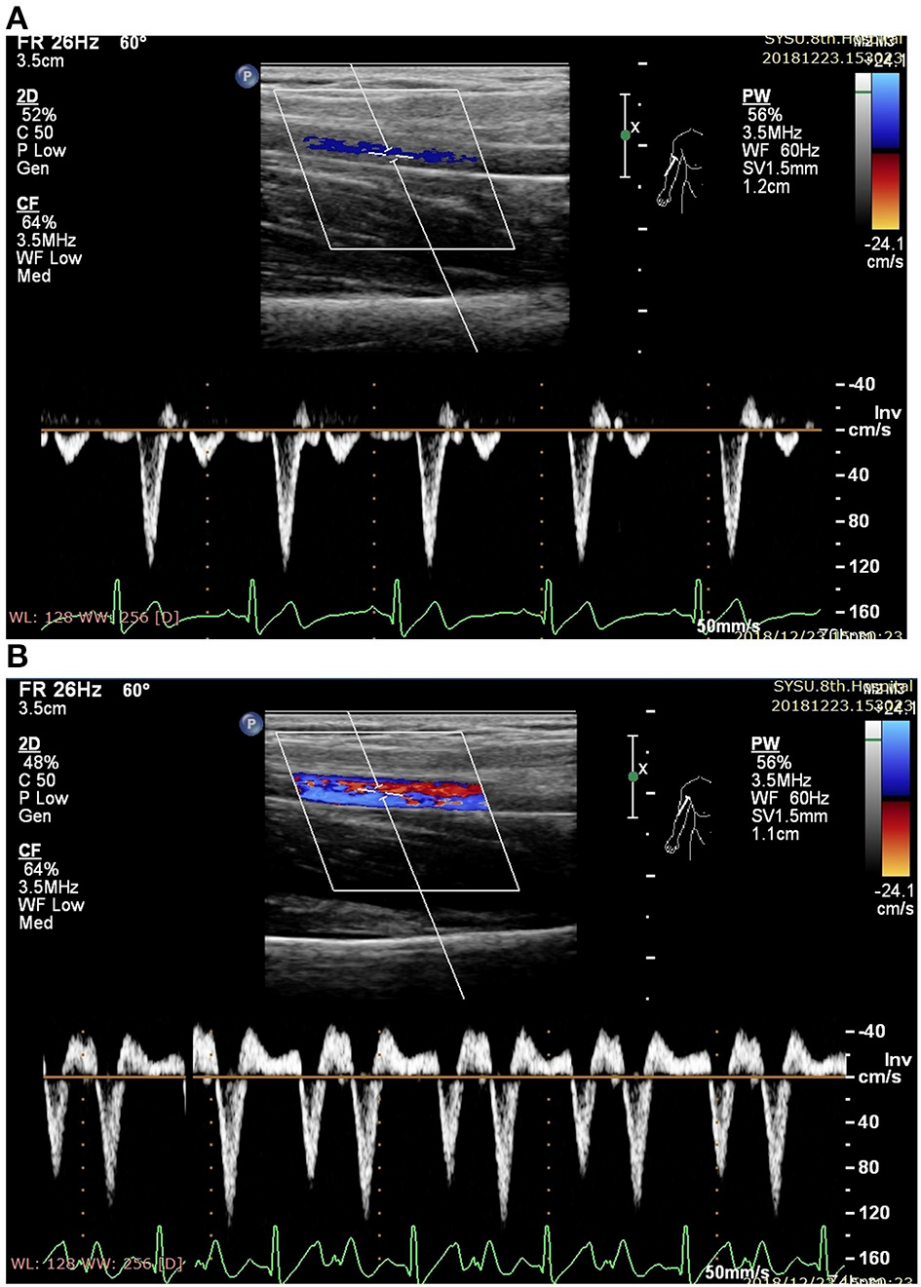
Measurement of blood flow velocity spectrum in the right brachial (RB) artery (**A** before EECP and **B** during EECP).

**Figure 3:**
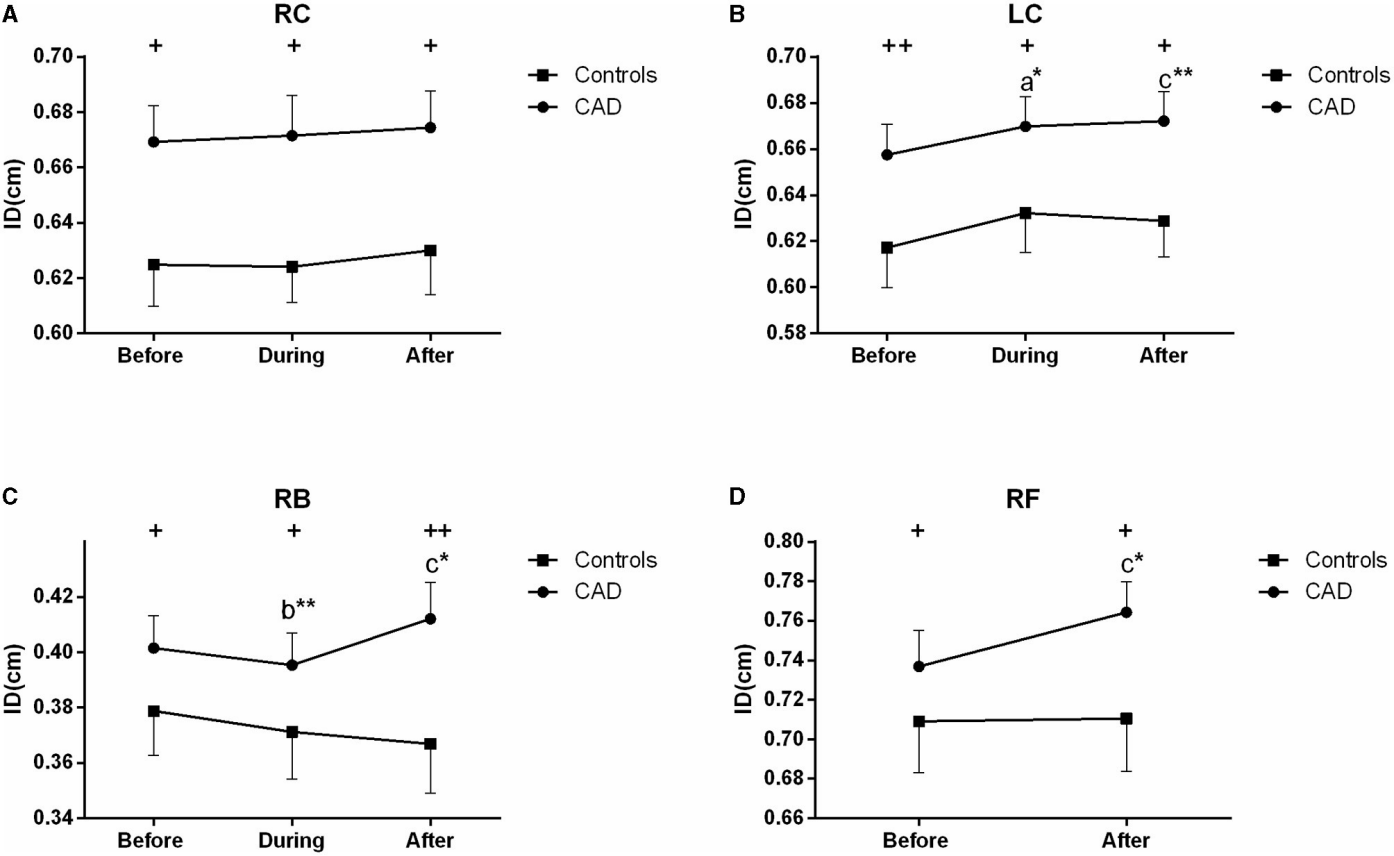
Effect of EECP on the mean inner diameters (ID) of the left and right carotid artery (RC/LC) **(A,B)**, RB **(C)**, and right femoral (RF) artery **(D)** in the patients with coronary artery disease (CAD) and controls before, during, and after a 45 min-EECP. a indicates significant differences in measurements obtained during EECP vs. pre-EECP; b indicates differences between post-EECP vs. during-EECP, and c refers to comparisons between post-EECP vs. pre-EECP. “^*^” and “^**^” denote *p* < 0.5 and < 0.01 respectively. “+” and “++” denote *p*-values of the difference between groups < 0.5 and < 0.01, respectively.

**Figure 4:**
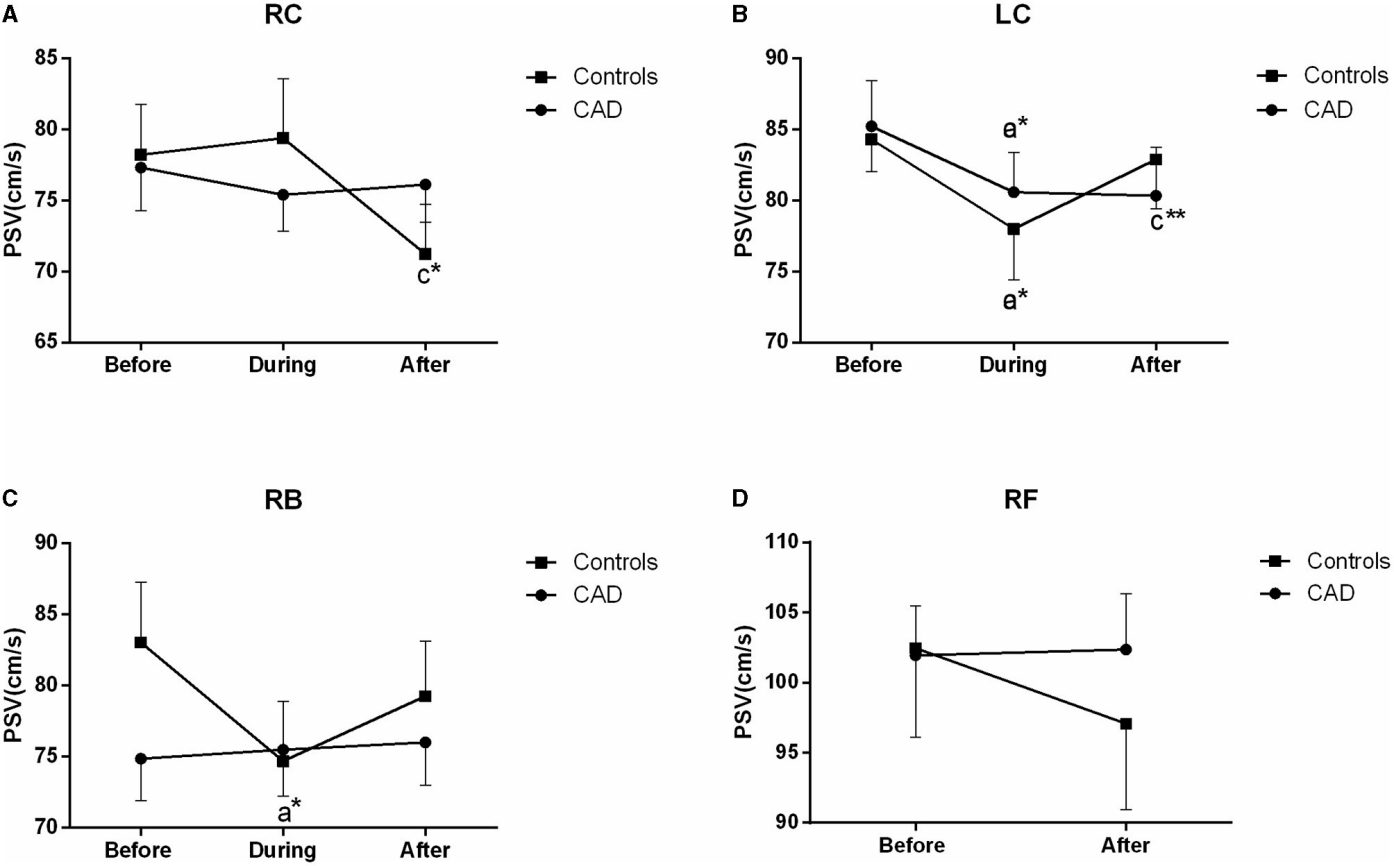
Effect of EECP on the peak systolic velocity (PSV) of the RC and LC **(A,B)**, RB **(C)**, and RF **(D)** in the patients with CAD and controls before, during, and after a 45 min-EECP. a indicates significant differences in measurements obtained during EECPvs. pre-EECP; c refers to comparisons between post-EECP vs. pre-EECP. ^*^ and ^**^ denote *p* < 0.5 and < 0.01 respectively.

**Figure 5:**
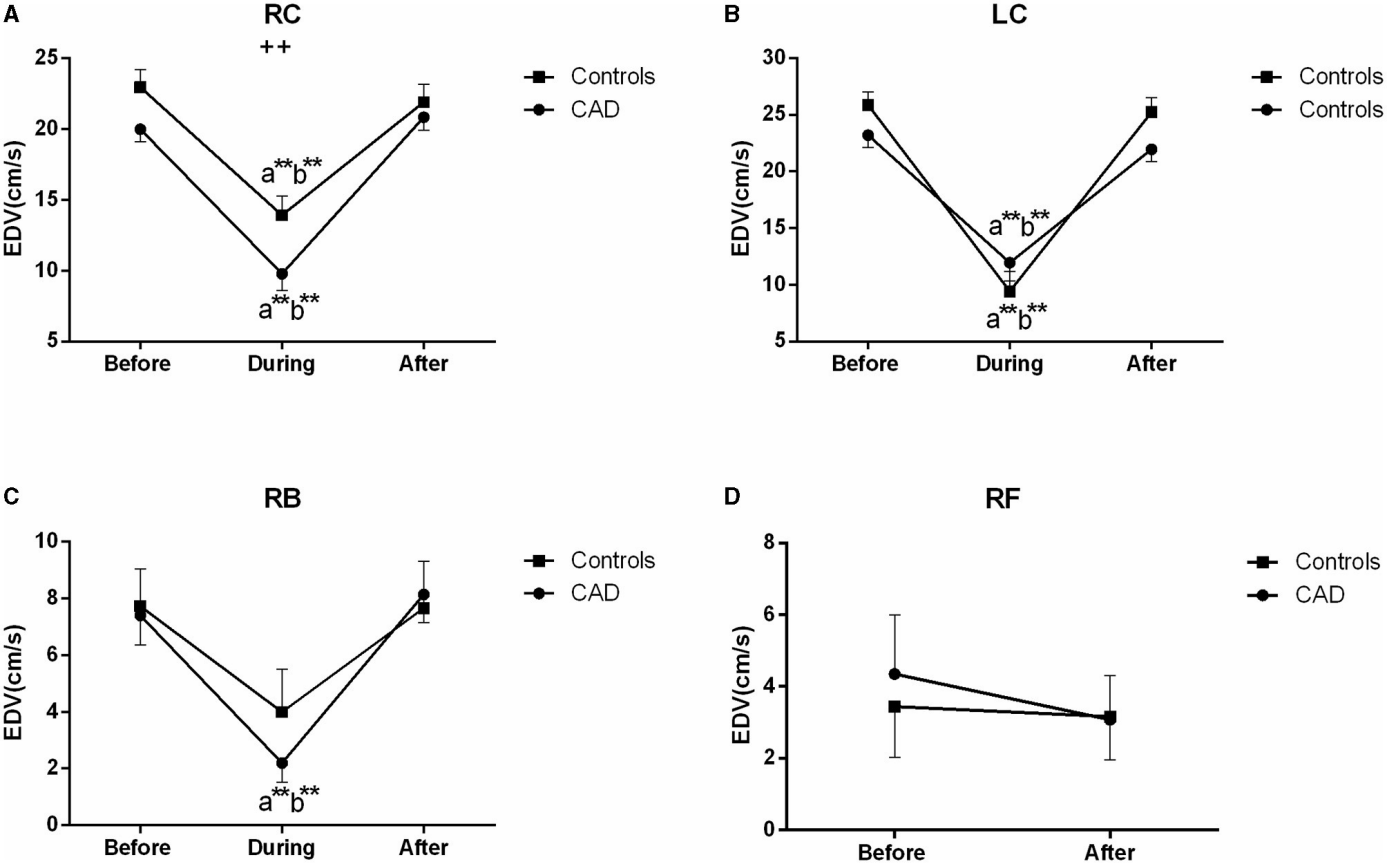
Effect of EECP on the end-diastolic velocity (EDV) of RC and LC **(A,B)**, RB **(C)**, and RF **(D)** in the patients with CAD and controls before, during, and after a 45 min-EECP. a indicates significant differences in measurements obtained during EECP vs. pre-EECP; b indicates differences between post-EECP vs. during-EECP. ^**^ and ^++^ denote *p*-values < 0.01 of the difference between groups.

**Figure 6:**
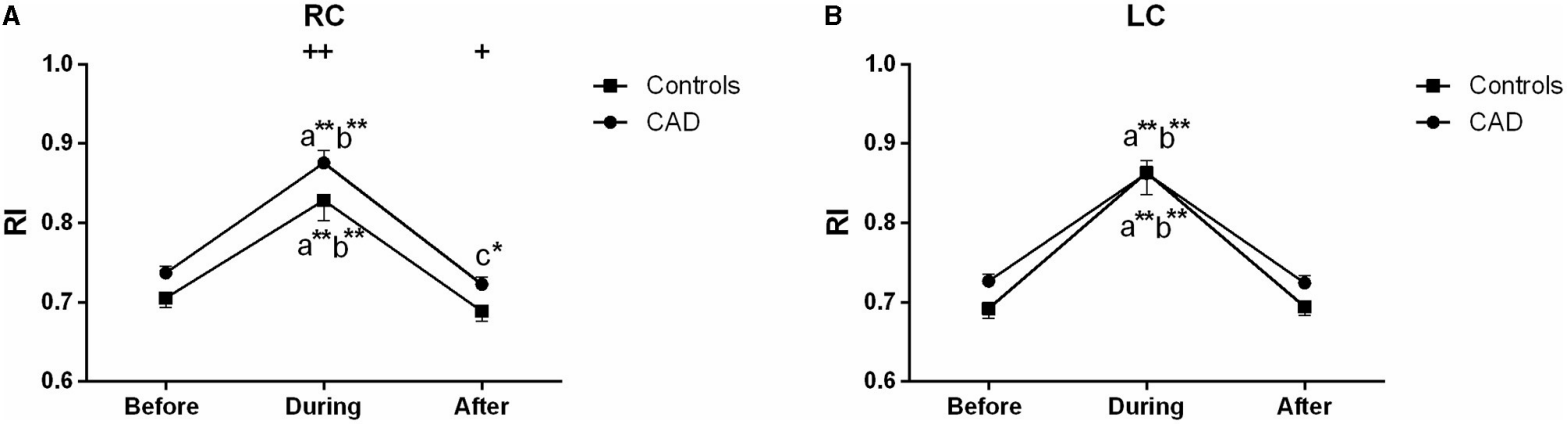
Effect of EECP on the resistance index (RI) of RC **(A)** and LC **(B)** in the patients with CAD and controls before, during, and after a 45 min-EECP. a indicates significant differences in measurements obtained during EECP vs. pre-EECP; b indicates differences between post-EECP vs. during-EECP, and c refers to comparisons between post-EECP vs. pre-EECP. ^*^ and ^**^ denote *p* < 0.5 and < 0.01 respectively. ^+^ and ^++^ denote *p*-values of the difference between groups < 0.5 and < 0.01, respectively.

**Figure 7:**
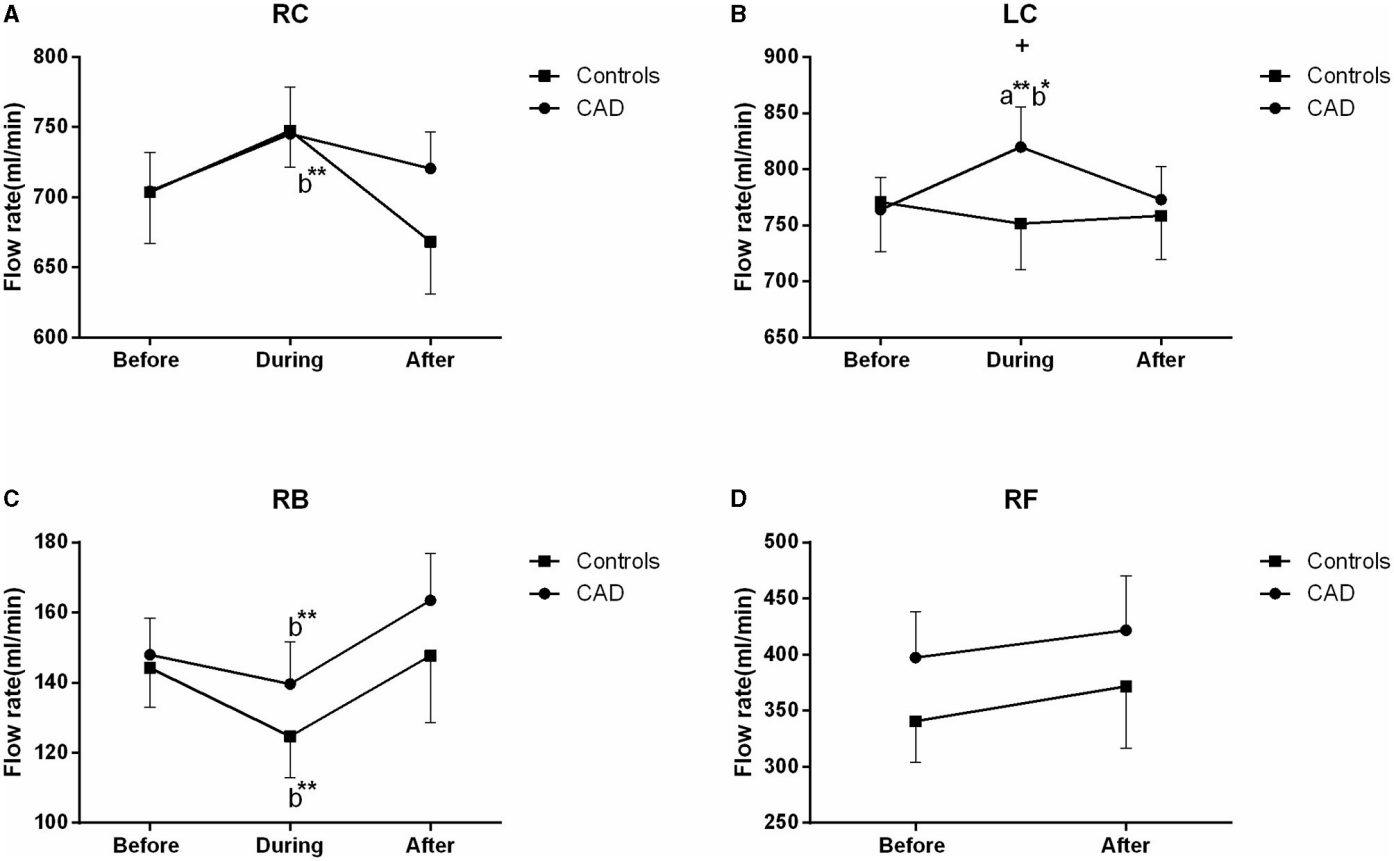
Effect of EECP on the flow rate (FR) of RC and LC **(A,B)**, RB **(C)**, and RF **(D)** in the patients with CAD and controls before, during, and after a 45 min-EECP. a indicates significant differences in measurements obtained during EECP vs. pre-EECP; b indicates differences between post-EECP vs. during-EECP. ^*^ and ^**^ denote *p* < 0.5 and < 0.01 respectively. ^+^ denotes *p*-values of the difference between groups < 0.5.

The ultrasound picture and Doppler spectrum of carotid and brachial arteries during EECP are presented in Figures 1, 2, respectively. The effect of EECP on the measured variables varied in each artery as did the differences between the patients with CAD and controls are illustrated in Figures 3–**7**. In addition, the significant differences in the hemodynamic parameters in females and males of both groups were analyzed by subgroup analysis. These results are shown in Table 2.

**Table 2:** Significant differences of the hemodynamic variables for the patients with CAD and controls by subgroup analysis.

**CAD**		**Female (*****n*** **=** **14)**			**Male (*****n*** **=** **28)**		
**Artery**	**Variable**	***Before***	***During***	***After***	***Before***	***During***	***After***
RC	RI	0.731 ± 0.014^*^	0.798 ± 0.023^**^	0.703 ± 0.016^**^	0.740 ± 0.010^**^	0.915 ± 0.017^**^	0.732 ± 0.010
LC	PSV (cm/s)	89.285 ± 4.446^**^	83.541 ± 4.060	86.018 ± 5.565	83.170 ± 4.243	79.083 ± 3.722	77.490 ± 4.217^**^
	FR (ml/min)	779.718 ± 50.483^*^	879.522 ± 60.419	824.476 ± 57.001	756.306 ± 34.921	790.222 ± 43.359	747.223 ± 33.274
RB	PSV (cm/s)	76.444 ± 6.871	71.140 ± 6.978^*^	81.191 ± 7.256	74.062 ± 2.838	77.663 ± 3.456	73.416 ± 2.736
	FR (ml/min)	138.667 ± 18.659	136.900 ± 26.098	165.519 ± 24.443	152.599 ± 12.840	140.961 ± 12.700^*^	162.473 ± 16.322
**Control**		**Female (*****n*** **=** **14)**			**Male (*****n*** **=** **7)**		
**Artery**	**Variable**	***Before***	***During***	***After***	***Before***	***During***	***After***
RB	FR (ml/min)	129.891 ± 11.601	100.755 ± 9.515	101.260 ± 11.246	179.977 ± 21.346	184.630 ± 18.061^*^	263.706 ± 21.276^*^
LC	RI	0.696 ± 0.007^**^	0.854 ± 0.030^**^	0.683 ± 0.014	0.682 ± 0.009^*^	0.887 ± 0.064^*^	0.723 ± 0.013^*^

*RC, LC, RB, and RF are the right carotid, left carotid, brachial and femoral arteries, respectively. PSV, peak systolic velocity; ID, mean inner diameter; FR, mean flow rate; EDV, end-diastolic velocity; RI, resistance index*.

* and ** * enote p < 0.5 and < 0.01 respectively*.

### Mean ID

During EECP, the ID of the LC was significantly increased compared with the baseline in the patients with CAD (*P* = 0.013, Figure 3B). Immediately after EECP, the ID of the LC, RB, and RF was significantly higher than that at the baseline (all *P* < 0.05) in the patients with CAD. In addition, the ID of the patients with CAD in the carotid, brachial and femoral arteries was also higher than that in the control groups (all *P* < 0.05, Figure 3). However, there was no significant difference in the ID of the controls during and immediately after EECP (all *P* > 0.05).

### PSV

The PSV of the LC was significantly reduced during (*P* = 0.015) and after EECP (*P* = 0.005) compared with the baseline in the patients with CAD. However, in the control group, the PSV in the RC and RB was significantly decreased after and during EECP, respectively. These results are illustrated in Figure 4. Based on the subgroup analysis of gender, there was a different pattern in the PSV of the LC and RB between males and females immediately after EECP (both *P* < 0.05, Table 2).

### EDV

The EDV in the CCAs significantly decreased in the two groups during EECP, and then markedly increased following the 45 min-EECP (all *P* < 0.01, Figure 5). Additionally, during EECP, the EDV of the RC was lower in the patients with CAD than in the controls (*P* = 0.000, Figure 5A). The EDV in the RB was significantly reduced compared with the baseline only in the patients with CAD (*P* = 0.000, Figure 5C). However, there was no significant difference in the EDV of the RF immediately after EECP in the two groups (*P* > 0.05, Figure 5D).

### RI

In the carotid arteries, there was a significant increase in the RI during EECP both in the patients and controls (Figure 6). However, in the RC, the effect was maintained when measured at the end of the treatment period only in the patients with CAD (*P* = 0.039, Figure 6A). Moreover, the baseline values of the patients were not significantly higher than those of the controls but differ in the RC during (*P* = 0.002) and immediately after EECP (*P* = 0.036, Figure 6A). Furthermore, by the subgroup analysis of gender, the RI in the RC was significantly lower immediately after EECP compared with the baseline only in females with CAD (0.703 ± 0.016 vs. 0.731 ± 0.014, *P* = 0.002, Table 2), while the same change in the RI of the LC was shown only in healthy men (0.723 ± 0.013 vs. 0.682 ± 0.009, *P* = 0.044, Table 2).

### Mean FR

The FR of the LC was significantly increased during EECP, and then markedly recovered to the baseline in the patients with CAD (both *P* < 0.01, Figure 7B). It led to a significant difference in the FR between the patients with CAD and controls during EECP (*P* = 0.029). The FR in the RC was significantly reduced immediately after EECP compared with during EECP only in the controls. Contralaterally, the FR in the RB was markedly higher immediately after EECP than that during EECP in both groups (all *P* < 0.01, Figure 7C). By the subgroup analysis, the FR of the LC was markedly elevated during EECP in the females with CAD (879.522 ± 60.419 vs. 779.718 ± 50.483, *P* = 0.021). By contrast, the FR of the RB was significantly increased in the healthy males and those with CAD during and immediately after EECP (all *P* < 0.05, Table 2).

## Discussion

This study was designed to investigate the responses of the carotid and peripheral artery vascular and blood flow characteristics to EECP, both during and after a 45 min-session EECP. During EECP, there were different responses of ID, PSV, and FR in the CCAs and RB. Additionally, immediately after EECP, the ID of the LC, RB, and RF was significantly higher, while the PSV of the LC and the RI of the RC were significantly lower than those at the baseline only in the patients with CAD. Moreover, the RI and FR of the carotid arteries were changed in females, while a significant difference in the FR of the RB was shown in males.

In this study, a significant effect on the ID in the LC was observed in the patients with CAD. Additionally, immediately after EECP, the ID of the carotid and peripheral arteries was significantly higher than that at the baseline only in the patients with CAD. Importantly, they were also higher than those in the control groups. Previous large-scale studies have reported that carotid artery response is closely associated with coronary heart disease (23, 24). In addition, a study supported the present findings that EECP had arterial effects on large and small vessels of the carotid circulation in patients with CAD (9). One possible mechanism is by decreasing arterial pressure (25), the other mechanism is EECP, as well as physical training, may also activate the catecholamines and/or metabolic vasodilator pathways (9). A study also demonstrated that femoral artery diameter and vascular shear stress were significantly increased after supervised exercise training (26). Studies have also shown that changes in shear rate led by femoral artery vascular tone elicit an increased femoral baseline diameter after EECP intervention (19). More importantly, studies have demonstrated that endothelial functional changes induced by the shear rate in the peripheral circulations were modestly correlated with changes in the coronary flow reserve (27). The acute increase of ID in the patients with CAD may be related to the effects of peripheral function on the coronary circulation in this study (28).

There were some controversies in the changes of ID in the RB and RF during and immediately after EECP. Zhang et al. found that EECP did not cause an increase in the internal diameter of the RB (29). Gurovich et al. reported that peak diameter was also not increased, while absolute change (mm) of the brachial and femoral arteries significantly increased immediately after EECP (19). They found that the change of the brachial artery diameter affected the improvement of the brachial flow-mediated dilation (FMD), which was closely related to an increase in the brachial artery shear stress (19). Studies have suggested that both flow- and pressure-induced forces played an important role in vessel wall diameter (30–32). Inflation of the EECP cuffs can produce high-pressure retrograde blood flow in the femoral arteries and simultaneous moderate-pressure antegrade flow in the brachial arteries (29, 33). The brachial blood flow velocity in the diastole increased by 132% and led to the increase of the brachial artery wall shear stress (29). Cai et al. observed a 1.2-fold increase in femoral artery retrograde blood flow velocity (34). In this study, the PSV of the patients with CAD was significantly reduced during and after EECP compared with the baseline. In addition, the PSV of the RB and EDV of the carotid and peripheral arteries also significantly decreased in the patients with CAD.

Although few studies have focused on blood flow velocity during EECP, the use of an intra-aortic balloon pump, which has some similar mechanisms of action to EECP, induced a decrease of carotid PSV, and an increase in mean blood flow velocity (35) which supports the findings that cerebral flow velocity in the systole is significantly decreased during EECP both in the patients and controls (36). This was attributed to the decreased systolic blood pressure and enhanced cerebral vascular autoregulation (36). Furthermore, decreased systolic blood pressure reduces cardiac afterload after deflation, resulting in decreased PSV. Immediately after EECP, there was no significant difference in the systolic flow velocity in both the patients and controls (36). By contrast, we found that the PSV and EDV of the LC and the RI of the RC were significantly lower than those at the baseline immediately after EECP. Some studies consisted of our findings that EECP increased the cerebral peak velocity in diastole both in the patients and controls (21, 36). It has been shown that improved carotid flow following EECP is associated with decreased arterial stiffness and resistance, dependent upon arterial pressure, modulation of smooth muscle activity, and shear stress (9). More importantly, other investigations suggested that a better coronary vasodilatory response was associated with well-functioning endothelium and blood flow velocity (28).

Some studies have found that blood flow velocity is a parameter related to the arterial diameter, RI, and blood flow (37). More importantly, studies also reported that the regulation of steady blood flow is associated with carotid diameters and blood flow velocity (38). In the present study, there were different responses from the vascular and blood flow characteristics in the CCAs and RB during and immediately after EECP. The FR of the LC was significantly increased during EECP in the patients with CAD. It led to a significant difference in the FR between the patients with CAD and controls during EECP. The FR in the RC was significantly reduced immediately after EECP compared with during EECP only in the controls. Contralaterally, the FR in the RB was markedly higher immediately after EECP than during EECP in both groups. There were some controversial results in the FR during and immediately after EECP. Levenson et al. (9) found that in patients with CAD, the mean carotid blood flow was significantly increased during EECP. Lin et al. found that the cerebral blood flow of patients with ischemic stroke increased during EECP, but there was no change in the elderly subjects (21). Werner et al. found that EECP induced an augmentation in the cerebral blood flow both in the patients and controls (36). Cerebral autoregulation plays an important role in guaranteeing the constancy of cerebral perfusion during EECP (39). The increased FR in the RB after EECP may be related to the increase of the brachial artery wall shear stress (29).

We also found that the FR in the LC was higher than in the RC during and after the 45 min-EECP. This result was consistent with previous findings that the prognostic power of the altered vascular and blood flow characteristics for cardiovascular events is greater in the right CCA than the left (13, 14). Furthermore, the FR of the LC was markedly elevated during EECP in females with CAD. By contrast, the FR in the RB was significantly increased in healthy males and those with CAD during and immediately after EECP. Studies found that cardiovascular function during exercise is also affected by gender (40, 41). Meanwhile, the blood pressure and total peripheral resistance during exercise are higher in women than in men. They thought that the vasodilatory capacity of active muscle groups plays an important role in the cardiovascular differences between women and men (42).

In the study, the RI of the RC was also significantly lower immediately after EECP compared with the baseline only in females with CAD. The decrease of the RI which is strongly associated with cerebrovascular resistance can predict the degree of atherosclerosis and is also beneficial to the increase of blood flow (42, 43). Although the RI significantly increased during EECP, it recovered quickly after the treatment stopped. More importantly, the RI of the right CCA was lower after treatment than the pre-EECP values in the patients with CAD. Our results support those of a previous study that short-term EECP reduces the β-stiffness index and carotid vascular resistance in patients with CAD (9). In addition, studies reported that EECP can reduce distal brain resistance and increase cerebral perfusion (21). It suggests that the benefits of EECP may be attributable to the global improvement of cerebral perfusion (44). Moreover, cerebral autoregulation confirms the constancy of cerebral blood flow under fluctuant cerebral perfusion pressure (39). Furthermore, decreased RI elevates peripheral vascular function, which may, in turn, induce an improvement of the diastolic flow pattern in the coronary arteries (45). Indeed, reduced RI is an important reason for the improvement of carotid hemodynamics in patients with CAD.

### Limitations

Some limitations of the present study should be emphasized. Firstly, the sample size was relatively small. Secondly, the order of the measurements after the EECP was femoral, brachial, RC, and LC artery to firstly observe the blood flow from the legs which was directly pressurized during and after EECP. Therefore, at the end of the treatment, the recovery time was minimal for the femoral and increased by 30 s−1 min for each subsequent measurement site. Thirdly, we did not continuously measure these arteries at 1–2 min after EECP. Further study is needed to investigate the duration of the effect on these muscular arteries.

## Conclusions

Enhanced external counterpulsation creates different acute responses from the inner diameters, blood flow velocity, and FR between the carotid and peripheral arteries, with more obvious effects in both carotid arteries. Additionally, after the 45-min EECP, the more sustained effects in the inner diameters, RI, blood flow velocity, and FR were shown only in the patients with CAD. Moreover, an acute improvement brought by EECP to the FR of the brachial artery and blood flow velocity of the carotid artery showed in men, while the FR and RI of carotid arteries changed in females. This indicated that EECP should be conducted with different counterpulsation modes, separately intervening carotid arteries and peripheral arteries. More importantly, these findings suggest that EECP can regulate the vascular and blood flow characteristics of conduit arteries, and further improve the carotid and peripheral function in patients with CAD.

## Data Availability Statement

The raw data supporting the conclusions of this article will be made available by the authors, without undue reservation.

## Ethics Statement

The studies involving human participants were reviewed and approved by Medical Ethics Committee of the Eighth Affiliated Hospital of Sun Yat-sen University. The patients/participants provided their written informed consent to participate in this study.

## Author Contributions

YZ and GW proposed the scientific problems. YZ, GW, and JD designed the experiments. YZ, ZM, ZC, WZ, and HW collected the experimental data. YZ and XZ processed and calculated the data. YZ conducted the statistical analysis and wrote the draft manuscript. WW and GW contributed to the revision and final version of the manuscript. All authors contributed to the article and approved the submitted version.

## Funding

This work was, in part, supported by the National Natural Science Foundation of China (Grant No. 819770367 and 81670417) and the National Key Research and Development Program of China (No. 2020YFC2004400).

## Conflict of Interest

The authors declare that the research was conducted in the absence of any commercial or financial relationships that could be construed as a potential conflict of interest.

## Publisher's Note

All claims expressed in this article are solely those of the authors and do not necessarily represent those of their affiliated organizations, or those of the publisher, the editors and the reviewers. Any product that may be evaluated in this article, or claim that may be made by its manufacturer, is not guaranteed or endorsed by the publisher.
